# Correspondence

**Published:** 1874-08

**Authors:** J. M. Tullis

**Affiliations:** Church Hill, Ga.


CHURCH HILL, GA., June 11, 1874.

*Messrs. Editors:*

I was called to visit Mrs. W., May 7, 1874, age about twenty-one years old, pregnant with her second child. After examination of the case, I found her laboring under anasarcal dropsy of the lower extremities and the lower portion of her bowels, with infiltration of her labiæ pudendiæ, with an effusion of waters, until the labiæ pudendiæ was swollen to such an extent that she could not walk or stand on her feet, or set up, and suffering with a great deal of pain from it. I tapped or scarrified her labiæ on the internal surface with a scarrificator, and drew off the water, which ran off profusely, which caused the parts to assuage down in a short time so that she could stand on her feet and walk about again. I then put her upon 20 grs. of calomel, and 20 grs. of rhei., and 4 grs. of podophyllin, made into 10 pills, and gave her three every third night in combination with some diuretic medicines.

On the 10th, I visited her again, and found her in the same condition as she was before. I then applied the same treatment as before, with the same result. I then visited her again on the night of the 15th, and found her in the same condition as before, and I continued the same treatment as before. I was again called to visit her on the 21st of May, at which time I found her in labor, and in a short time I delivered her of a living female child of medium size, without any difficulty in the case.

On the 22nd of May, 1872, I delivered this same woman of her first child by craniotomy. She is doing well at this time, and is able to attend to her domestic affairs, etc.

I report you this case, Messrs. Editors, which you are at liberty to dispose of as you may think proper.

Respectfully yours,

J. M. Tullis, M.D.

